# Di-μ-acetato-κ^4^ *O*:*O*-bis­{(acetato-κ^2^ *O*,*O*′)bis­[3-(1*H*-imidazol-1-yl-κ*N* ^3^)-1-phenyl­propan-1-one]cadmium} tetra­hydrate

**DOI:** 10.1107/S1600536811050021

**Published:** 2011-11-30

**Authors:** Jian-Hua Guo

**Affiliations:** a College of Chemistry, Tianjin Key Laboratory of Structure and Performance for Functional Molecules, Tianjin Normal University, Tianjin 300387, People’s Republic of China

## Abstract

In the mol­ecular structure of the title neutral binuclear complex, [Cd_2_(C_2_H_3_O_2_)_4_(C_12_H_12_N_2_O)_4_]·4H_2_O, each Cd^II^ atom is six-coordinated and exhibits a distorted octa­hedral geometry. Three O atoms from two acetate ions and one monodentate 3-(1*H*-imidazol-1-yl-κ*N* ^3^)-1-phenyl­propan-1-one (*L*) ligand form the equatorial plane, while the bridging-O atom forming the longer Cd—O distance,and the N atom of the second *L* ligand, forming the longer Cd—N distance, occupy axial positions with an N—Cd—O angle of 170.77 (7)°. Inter­molecular O—H⋯O hydrogen bonds exist between the lattice water mol­ecules and the acetate ions of adjacent mol­ecules, resulting in a two-dimensional supra­molecular structure.

## Related literature

For reviews on the generation of supra­molecular structures based on coordination complexes, see: Barnett & Champness (2003 ▶); Roesky & Andruh (2003 ▶); Zaworotko (2001 ▶). 
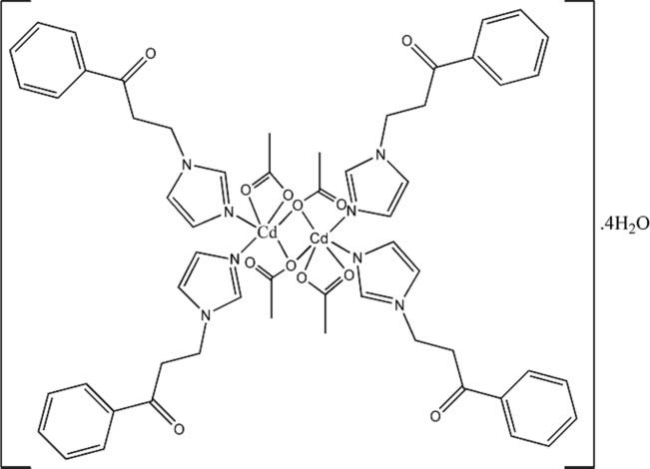


## Experimental

### 

#### Crystal data

[Cd_2_(C_2_H_3_O_2_)_4_(C_12_H_12_N_2_O)_4_]·4H_2_O
*M* *_r_* = 1333.98
Monoclinic, 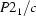

*a* = 18.818 (3) Å
*b* = 10.6490 (17) Å
*c* = 15.864 (3) Å
β = 112.036 (2)°
*V* = 2946.8 (8) Å^3^
*Z* = 2
Mo *K*α radiation
μ = 0.80 mm^−1^
*T* = 296 K
0.28 × 0.22 × 0.20 mm

#### Data collection

Bruker APEXII CCD area-detector diffractometer
Absorption correction: multi-scan (*SADABS*; Sheldrick, 1996 ▶) *T*_min_ = 0.808, *T*_max_ = 0.857
17422 measured reflections
6806 independent reflections
4666 reflections with *I* > 2σ(*I*)
*R*_int_ = 0.027

#### Refinement

*R*[*F* ^2^ > 2σ(*F* ^2^)] = 0.031
*wR*(*F* ^2^) = 0.077
*S* = 1.00
6806 reflections
372 parameters
1 restraint
H-atom parameters constrained
Δρ_max_ = 0.53 e Å^−3^
Δρ_min_ = −0.46 e Å^−3^

### 

Data collection: *APEX2* (Bruker, 2003 ▶); cell refinement: *SAINT* (Bruker, 2003 ▶); data reduction: *SAINT*; program(s) used to solve structure: *SHELXS97* (Sheldrick, 2008 ▶); program(s) used to refine structure: *SHELXL97* (Sheldrick, 2008 ▶); molecular graphics: *SHELXTL* (Sheldrick, 2008 ▶) and *DIAMOND* (Brandenburg & Berndt, 1999 ▶); software used to prepare material for publication: *SHELXTL*.

## Supplementary Material

Crystal structure: contains datablock(s) global, I. DOI: 10.1107/S1600536811050021/mw2039sup1.cif

Structure factors: contains datablock(s) I. DOI: 10.1107/S1600536811050021/mw2039Isup2.hkl

Additional supplementary materials:  crystallographic information; 3D view; checkCIF report

## Figures and Tables

**Table 1:** Selected bond lengths (Å)

Cd1—O5^i^	2.2331 (18)
Cd1—N1	2.238 (2)
Cd1—N3	2.349 (2)
Cd1—O3	2.354 (2)
Cd1—O4	2.446 (2)
Cd1—O5	2.5855 (18)

Symmetry code: (i) 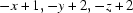.

**Table 2:** Hydrogen-bond geometry (Å, °)

*D*—H⋯*A*	*D*—H	H⋯*A*	*D*⋯*A*	*D*—H⋯*A*
O7—H7*A*⋯O3^ii^	0.85	2.11	2.949 (3)	169
O7—H7*B*⋯O4^iii^	0.85	1.99	2.829 (3)	171
O8—H8*B*⋯O6^iii^	0.85	2.03	2.838 (3)	159
O8—H8*A*⋯O7^iv^	0.85	1.97	2.818 (3)	174

Symmetry codes: (ii) 
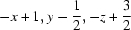
; (iii) 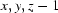; (iv) 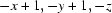.

**Fig. 1.**
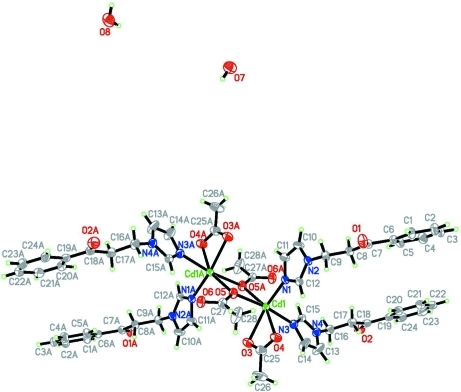
The molecular structure of (I) using 30% probability ellipsoids (Symmetry transformation used to generate equivalent atoms: -x+1,-y+2,-z+2).

**Fig. 2.**
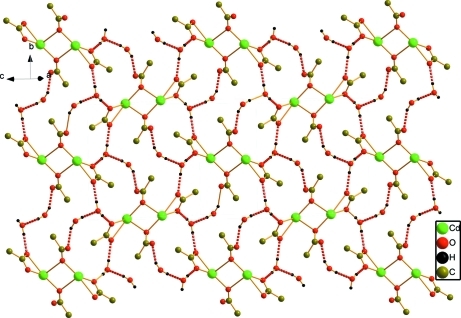
The 2-D supramolecular framework structure of compound, showing O—H···O hydrogen bonds as red dashed lines (the L ligands have been removed for clarity).

## supplementary crystallographic information

### Comment

Neutral organic ligands containing rigid or flexible spacers, such as 4,4'-bipyridine, 1,2-bis(4'-pyridyl)ethane, 1,2-bis(4-pyridyl)propane and many others, have been used to generate a rich variety of metal-organic architectures with different metal ions by various reaction procedures (Barnett & Champness, 2003; Zaworotko, 2001; Roesky & Andruh, 2003). In our recent research, we have initiated a synthetic approach employing the ligand 3-(1*H*-imidazol-1-yl)-1-phenylpropan-1-one (*L*), which consists of a propanone unit substituted with an imidazole and a phenyl group, in reaction with different metal ions to construct new functional frameworks. To explore this series, we synthesized the title compound, (I), a new Cd^II^ complex based on *L*. In the molecular structure of (I) (Fig. 1) each Cd^II^ atom is six-coordinated and exhibits a distorted octahedral geometry. Three O atoms from two acetate ions and one monodentate *L* ligand form the equatorial plane while one O atom from one acetate ion and the other monodentate *L* ligand occupy axial positions with an N3—Cd1—O5 angle of 170.77 (7)°.  Atoms O5 and O5A of one pair of acetate ions serve to bridge the Cd^II^ centers in the centrosymmetric binuclear units. The mean planes of the imidazole and phenyl rings in the two unique ligands are nearly perpendicular to one another with the angles between these planes being 78.6 (1)° and 76.2 (1)°, respectively. Analysis of the crystal packing indicates that intermolecular O—H···O hydrogen bonds involving the lattice water molecules and both types of actate ions extend the binuclear units to produce a 2-D supramolecular framework structure, as shown in Fig. 2.

### Experimental

Cd(OAc)_2_.2H_2_O (26.7 mg, 0.1 mmol) and 3-(1*H*-imidazol-1-yl)-1-phenylpropan-1-one (22.2 mg, 0.1 mmol) were mixed in a CH_3_CN/H_2_O (20 ml, 1:1 *v*/*v*) solution with vigorous stirring for *ca* 30 min. The resulting solution was filtered and left to stand at room temperature. Colorless block crystals of (I) suitable for X-ray analysis were obtained in 75% yield by slow evaporation of the solvent over a period of 1 week. Analysis, calculated for CdC_28_H_34_N_4_O_8_: C 50.42, H 5.14, N 8.40; found: C 50.45, H 5.03, N 8.32.

### Refinement

Although all H atoms were visible in difference maps, they were placed in geometrically calculated positions, with C—H and O—H distances in the range 0.93–0.97Å and 0.85 Å, respectively, and included in the final refinement in the riding model approximation, with *U*_iso_(H) = 1.2*U*_eq_(C) for aromatic and methylene H atoms. and *U*_iso_(H) = 1.5*U*_eq_(C or O) for methyl and H_2_O H atoms

### Crystal data

**Table:**

[Cd_2_(C_2_H_3_O_2_)_4_(C_12_H_12_N_2_O)_4_]·4H_2_O	*F*(000) = 1368
*M**_r_* = 1333.98	*D*_x_ = 1.503 Mg m^−^^3^
Monoclinic, *P*2_1_/*c*	Mo *K*α radiation, λ = 0.71073 Å
Hall symbol: -P 2ybc	Cell parameters from 4777 reflections
*a* = 18.818 (3) Å	θ = 2.6–25.4°
*b* = 10.6490 (17) Å	µ = 0.80 mm^−^^1^
*c* = 15.864 (3) Å	*T* = 296 K
β = 112.036 (2)°	Block, colorless
*V* = 2946.8 (8) Å^3^	0.28 × 0.22 × 0.20 mm
*Z* = 2	

### Data collection

**Table:**

Bruker APEXII CCD area-detector diffractometer	6806 independent reflections
Radiation source: fine-focus sealed tube	4666 reflections with *I* > 2σ(*I*)
graphite	*R*_int_ = 0.027
phi and ω scans	θ_max_ = 27.9°, θ_min_ = 2.2°
Absorption correction: multi-scan (*SADABS*; Sheldrick, 1996)	*h* = −24→24
*T*_min_ = 0.808, *T*_max_ = 0.857	*k* = −13→7
17422 measured reflections	*l* = −19→20

### Refinement

**Table:**

Refinement on *F*^2^	Primary atom site location: structure-invariant direct methods
Least-squares matrix: full	Secondary atom site location: difference Fourier map
*R*[*F*^2^ > 2σ(*F*^2^)] = 0.031	Hydrogen site location: inferred from neighbouring sites
*wR*(*F*^2^) = 0.077	H-atom parameters constrained
*S* = 1.00	*w* = 1/[σ^2^(*F*_o_^2^) + (0.0307*P*)^2^ + 1.0868*P*] where *P* = (*F*_o_^2^ + 2*F*_c_^2^)/3
6806 reflections	(Δ/σ)_max_ = 0.004
372 parameters	Δρ_max_ = 0.53 e Å^−^^3^
1 restraint	Δρ_min_ = −0.46 e Å^−^^3^

### Special details

**Table:**

Geometry. All e.s.d.'s (except the e.s.d. in the dihedral angle between two l.s. planes) are estimated using the full covariance matrix. The cell e.s.d.'s are taken into account individually in the estimation of e.s.d.'s in distances, angles and torsion angles; correlations between e.s.d.'s in cell parameters are only used when they are defined by crystal symmetry. An approximate (isotropic) treatment of cell e.s.d.'s is used for estimating e.s.d.'s involving l.s. planes.
Refinement. Refinement of *F*^2^ against ALL reflections. The weighted *R*-factor *wR* and goodness of fit *S* are based on *F*^2^, conventional *R*-factors *R* are based on *F*, with *F* set to zero for negative *F*^2^. The threshold expression of *F*^2^ > σ(*F*^2^) is used only for calculating *R*-factors(gt) *etc*. and is not relevant to the choice of reflections for refinement. *R*-factors based on *F*^2^ are statistically about twice as large as those based on *F*, and *R*- factors based on ALL data will be even larger.

### Fractional atomic coordinates and isotropic or equivalent isotropic displacement parameters (Å^2^)

**Table:**

	*x*	*y*	*z*	*U*_iso_*/*U*_eq_	
Cd1	0.421249 (10)	0.990415 (18)	1.052021 (12)	0.03958 (7)	
O1	0.08650 (12)	0.52281 (17)	0.69389 (16)	0.0617 (6)	
O2	0.09479 (14)	1.4665 (2)	1.14769 (16)	0.0664 (6)	
O3	0.50729 (12)	0.9464 (2)	1.20070 (14)	0.0602 (5)	
O4	0.41427 (12)	0.80882 (18)	1.14306 (14)	0.0577 (5)	
O5	0.51810 (10)	0.86982 (17)	1.00202 (13)	0.0477 (5)	
O6	0.62749 (11)	0.76847 (17)	1.04134 (15)	0.0548 (5)	
O7	0.37028 (13)	0.5542 (2)	0.13904 (16)	0.0776 (7)	
H7A	0.4013	0.5150	0.1847	0.116*	
H7B	0.3785	0.6323	0.1363	0.116*	
O8	0.69937 (13)	0.5343 (2)	0.04085 (17)	0.0788 (7)	
H8A	0.6793	0.5019	−0.0118	0.118*	
H8B	0.6753	0.5955	0.0522	0.118*	
N1	0.33866 (12)	0.9077 (2)	0.92267 (14)	0.0397 (5)	
N2	0.25575 (12)	0.7856 (2)	0.82184 (15)	0.0411 (5)	
N3	0.33891 (13)	1.0771 (2)	1.11710 (16)	0.0458 (5)	
N4	0.26263 (13)	1.2047 (2)	1.15386 (16)	0.0478 (6)	
C1	−0.07134 (16)	0.5507 (3)	0.6383 (2)	0.0521 (7)	
H1	−0.0525	0.4736	0.6285	0.062*	
C2	−0.14918 (18)	0.5682 (3)	0.6113 (2)	0.0621 (9)	
H2	−0.1825	0.5029	0.5833	0.075*	
C3	−0.17788 (17)	0.6812 (3)	0.6253 (2)	0.0575 (8)	
H3	−0.2305	0.6925	0.6070	0.069*	
C4	−0.12871 (17)	0.7779 (3)	0.6664 (2)	0.0585 (8)	
H4	−0.1482	0.8548	0.6754	0.070*	
C5	−0.05019 (16)	0.7610 (3)	0.6943 (2)	0.0506 (7)	
H5	−0.0171	0.8264	0.7228	0.061*	
C6	−0.02075 (15)	0.6472 (2)	0.68013 (18)	0.0404 (6)	
C7	0.06321 (15)	0.6234 (2)	0.70880 (18)	0.0412 (6)	
C8	0.11922 (14)	0.7264 (2)	0.75563 (18)	0.0421 (6)	
H8A'	0.1106	0.7533	0.8094	0.051*	
H8B'	0.1104	0.7979	0.7151	0.051*	
C9	0.20162 (15)	0.6829 (2)	0.7829 (2)	0.0460 (7)	
H9A	0.2116	0.6157	0.8271	0.055*	
H9B	0.2092	0.6499	0.7299	0.055*	
C10	0.27050 (18)	0.8835 (3)	0.77606 (19)	0.0588 (8)	
H10	0.2492	0.8969	0.7136	0.071*	
C11	0.32162 (17)	0.9573 (3)	0.8380 (2)	0.0535 (8)	
H11	0.3423	1.0308	0.8253	0.064*	
C12	0.29864 (15)	0.8039 (3)	0.91023 (18)	0.0435 (6)	
H12	0.2998	0.7497	0.9567	0.052*	
C13	0.2737 (2)	1.1008 (3)	1.2069 (3)	0.0884 (14)	
H13	0.2532	1.0861	1.2511	0.106*	
C14	0.3193 (2)	1.0238 (3)	1.1839 (3)	0.0791 (12)	
H14	0.3355	0.9452	1.2095	0.095*	
C15	0.30280 (15)	1.1856 (3)	1.10107 (19)	0.0456 (7)	
H15	0.3050	1.2428	1.0578	0.055*	
C16	0.21135 (17)	1.3088 (3)	1.1499 (2)	0.0533 (7)	
H16A	0.2235	1.3793	1.1189	0.064*	
H16B	0.2189	1.3352	1.2112	0.064*	
C17	0.12822 (16)	1.2713 (3)	1.1001 (2)	0.0529 (7)	
H17A	0.1193	1.2571	1.0366	0.063*	
H17B	0.1188	1.1928	1.1252	0.063*	
C18	0.07216 (17)	1.3690 (3)	1.10664 (19)	0.0496 (7)	
C19	−0.01133 (18)	1.3416 (3)	1.0617 (2)	0.0528 (8)	
C20	−0.03927 (19)	1.2307 (4)	1.0159 (2)	0.0643 (9)	
H20	−0.0052	1.1694	1.0127	0.077*	
C21	−0.1179 (2)	1.2100 (4)	0.9745 (2)	0.0803 (11)	
H21	−0.1363	1.1349	0.9442	0.096*	
C22	−0.1684 (2)	1.3016 (6)	0.9789 (3)	0.0948 (15)	
H22	−0.2209	1.2887	0.9508	0.114*	
C23	−0.1412 (3)	1.4115 (5)	1.0246 (3)	0.0941 (15)	
H23	−0.1755	1.4725	1.0278	0.113*	
C24	−0.0635 (2)	1.4325 (4)	1.0659 (2)	0.0701 (10)	
H24	−0.0456	1.5074	1.0967	0.084*	
C25	0.4710 (2)	0.8497 (3)	1.2087 (2)	0.0580 (8)	
C26	0.4947 (2)	0.7839 (4)	1.2998 (2)	0.0951 (13)	
H26A	0.4505	0.7716	1.3152	0.143*	
H26B	0.5316	0.8344	1.3456	0.143*	
H26C	0.5170	0.7040	1.2962	0.143*	
C27	0.55988 (16)	0.7733 (2)	1.03291 (19)	0.0414 (6)	
C28	0.5239 (2)	0.6615 (3)	1.0586 (3)	0.0913 (14)	
H28A	0.5395	0.5864	1.0368	0.137*	
H28B	0.4691	0.6690	1.0319	0.137*	
H28C	0.5402	0.6574	1.1236	0.137*	

### Atomic displacement parameters (Å^2^)

**Table:**

	*U*^11^	*U*^22^	*U*^33^	*U*^12^	*U*^13^	*U*^23^
Cd1	0.03298 (11)	0.04915 (12)	0.03321 (10)	−0.00241 (9)	0.00850 (8)	−0.00217 (9)
O1	0.0458 (12)	0.0424 (11)	0.0913 (17)	−0.0026 (9)	0.0192 (12)	−0.0128 (11)
O2	0.0781 (16)	0.0553 (13)	0.0701 (15)	0.0154 (11)	0.0328 (13)	−0.0027 (12)
O3	0.0589 (14)	0.0645 (13)	0.0470 (10)	0.0096 (11)	0.0079 (9)	0.0076 (10)
O4	0.0689 (15)	0.0505 (12)	0.0467 (12)	0.0119 (10)	0.0136 (11)	0.0053 (10)
O5	0.0434 (12)	0.0385 (10)	0.0631 (13)	0.0043 (8)	0.0221 (10)	0.0011 (9)
O6	0.0438 (12)	0.0479 (11)	0.0779 (15)	0.0059 (9)	0.0289 (11)	0.0033 (10)
O7	0.0796 (17)	0.0774 (15)	0.0695 (16)	−0.0069 (13)	0.0209 (13)	−0.0040 (13)
O8	0.0659 (16)	0.0728 (15)	0.0851 (18)	0.0276 (12)	0.0138 (13)	−0.0040 (13)
N1	0.0307 (12)	0.0490 (13)	0.0354 (12)	−0.0030 (10)	0.0078 (10)	−0.0028 (10)
N2	0.0321 (12)	0.0442 (12)	0.0399 (12)	−0.0049 (9)	0.0055 (10)	−0.0018 (10)
N3	0.0411 (14)	0.0507 (13)	0.0474 (14)	0.0069 (11)	0.0187 (11)	0.0059 (11)
N4	0.0452 (14)	0.0506 (13)	0.0537 (14)	0.0103 (11)	0.0254 (12)	0.0086 (12)
C1	0.0436 (17)	0.0450 (15)	0.063 (2)	−0.0082 (13)	0.0152 (15)	−0.0032 (14)
C2	0.0427 (19)	0.069 (2)	0.069 (2)	−0.0175 (16)	0.0144 (16)	−0.0054 (18)
C3	0.0347 (17)	0.082 (2)	0.0534 (19)	−0.0001 (16)	0.0144 (14)	0.0067 (17)
C4	0.0493 (19)	0.0628 (19)	0.061 (2)	0.0084 (15)	0.0184 (16)	−0.0030 (16)
C5	0.0412 (17)	0.0498 (16)	0.0554 (18)	−0.0030 (13)	0.0118 (14)	−0.0066 (14)
C6	0.0377 (15)	0.0437 (14)	0.0388 (15)	−0.0051 (12)	0.0131 (12)	0.0006 (12)
C7	0.0401 (16)	0.0377 (14)	0.0431 (15)	−0.0042 (11)	0.0126 (13)	0.0014 (12)
C8	0.0372 (15)	0.0396 (14)	0.0453 (16)	−0.0041 (11)	0.0106 (13)	−0.0029 (12)
C9	0.0390 (16)	0.0398 (14)	0.0528 (17)	−0.0060 (12)	0.0101 (13)	−0.0069 (13)
C10	0.069 (2)	0.0658 (19)	0.0327 (15)	−0.0226 (16)	0.0082 (15)	0.0039 (14)
C11	0.0566 (19)	0.0522 (17)	0.0440 (16)	−0.0187 (14)	0.0101 (14)	0.0048 (14)
C12	0.0349 (15)	0.0516 (16)	0.0396 (15)	−0.0042 (12)	0.0091 (12)	0.0055 (12)
C13	0.105 (3)	0.082 (3)	0.117 (3)	0.042 (2)	0.087 (3)	0.050 (2)
C14	0.092 (3)	0.067 (2)	0.109 (3)	0.033 (2)	0.072 (3)	0.041 (2)
C15	0.0405 (16)	0.0544 (17)	0.0425 (16)	0.0034 (13)	0.0164 (13)	0.0082 (13)
C16	0.056 (2)	0.0467 (16)	0.062 (2)	0.0089 (14)	0.0275 (16)	0.0016 (14)
C17	0.0543 (19)	0.0532 (17)	0.0546 (18)	0.0115 (14)	0.0244 (15)	−0.0007 (14)
C18	0.058 (2)	0.0533 (17)	0.0430 (16)	0.0172 (15)	0.0255 (15)	0.0116 (14)
C19	0.0548 (19)	0.068 (2)	0.0425 (17)	0.0206 (16)	0.0257 (15)	0.0180 (15)
C20	0.051 (2)	0.095 (3)	0.0479 (19)	0.0126 (19)	0.0204 (16)	0.0090 (18)
C21	0.060 (2)	0.129 (3)	0.050 (2)	−0.001 (2)	0.0185 (18)	0.009 (2)
C22	0.053 (2)	0.179 (5)	0.055 (2)	0.022 (3)	0.023 (2)	0.046 (3)
C23	0.073 (3)	0.147 (4)	0.077 (3)	0.060 (3)	0.046 (3)	0.057 (3)
C24	0.069 (3)	0.087 (2)	0.067 (2)	0.0357 (19)	0.040 (2)	0.0285 (19)
C25	0.066 (2)	0.0524 (19)	0.0524 (19)	0.0215 (16)	0.0184 (18)	0.0097 (15)
C26	0.116 (3)	0.083 (3)	0.058 (2)	0.007 (2)	0.001 (2)	0.029 (2)
C27	0.0458 (17)	0.0341 (13)	0.0492 (16)	−0.0003 (12)	0.0233 (14)	−0.0051 (12)
C28	0.097 (3)	0.0394 (18)	0.172 (4)	0.0031 (18)	0.090 (3)	0.010 (2)

### Geometric parameters (Å, °)

**Table:**

Cd1—O5^i^	2.2331 (18)	C7—C8	1.509 (3)
Cd1—N1	2.238 (2)	C8—C9	1.518 (3)
Cd1—N3	2.349 (2)	C8—H8A'	0.9700
Cd1—O3	2.354 (2)	C8—H8B'	0.9700
Cd1—O4	2.446 (2)	C9—H9A	0.9700
Cd1—O5	2.5855 (18)	C9—H9B	0.9700
Cd1—C25	2.749 (3)	C10—C11	1.342 (4)
O1—C7	1.214 (3)	C10—H10	0.9300
O2—C18	1.215 (3)	C11—H11	0.9300
O3—C25	1.268 (4)	C12—H12	0.9300
O4—C25	1.257 (4)	C13—C14	1.334 (4)
O5—C27	1.275 (3)	C13—H13	0.9300
O5—Cd1^i^	2.2331 (18)	C14—H14	0.9300
O6—C27	1.229 (3)	C15—H15	0.9300
O7—H7A	0.8500	C16—C17	1.517 (4)
O7—H7B	0.8499	C16—H16A	0.9700
O8—H8A	0.8500	C16—H16B	0.9700
O8—H8B	0.8500	C17—C18	1.512 (4)
N1—C12	1.311 (3)	C17—H17A	0.9700
N1—C11	1.364 (3)	C17—H17B	0.9700
N2—C12	1.344 (3)	C18—C19	1.490 (4)
N2—C10	1.358 (3)	C19—C20	1.382 (5)
N2—C9	1.464 (3)	C19—C24	1.398 (4)
N3—C15	1.316 (3)	C20—C21	1.393 (4)
N3—C14	1.369 (4)	C20—H20	0.9300
N4—C15	1.338 (3)	C21—C22	1.382 (6)
N4—C13	1.359 (4)	C21—H21	0.9300
N4—C16	1.455 (3)	C22—C23	1.371 (6)
C1—C2	1.376 (4)	C22—H22	0.9300
C1—C6	1.388 (4)	C23—C24	1.379 (5)
C1—H1	0.9300	C23—H23	0.9300
C2—C3	1.371 (4)	C24—H24	0.9300
C2—H2	0.9300	C25—C26	1.514 (4)
C3—C4	1.374 (4)	C26—H26A	0.9600
C3—H3	0.9300	C26—H26B	0.9600
C4—C5	1.386 (4)	C26—H26C	0.9600
C4—H4	0.9300	C27—C28	1.499 (4)
C5—C6	1.386 (4)	C28—H28A	0.9600
C5—H5	0.9300	C28—H28B	0.9600
C6—C7	1.492 (4)	C28—H28C	0.9600
			
O5^i^—Cd1—N1	100.91 (7)	C11—C10—N2	107.0 (2)
O5^i^—Cd1—N3	115.03 (7)	C11—C10—H10	126.5
N1—Cd1—N3	102.24 (8)	N2—C10—H10	126.5
O5^i^—Cd1—O3	105.01 (8)	C10—C11—N1	109.5 (2)
N1—Cd1—O3	145.14 (8)	C10—C11—H11	125.2
N3—Cd1—O3	87.54 (8)	N1—C11—H11	125.2
O5^i^—Cd1—O4	154.40 (7)	N1—C12—N2	111.5 (2)
N1—Cd1—O4	93.31 (8)	N1—C12—H12	124.3
N3—Cd1—O4	82.00 (7)	N2—C12—H12	124.3
O3—Cd1—O4	54.55 (8)	C14—C13—N4	107.2 (3)
O5^i^—Cd1—O5	72.10 (7)	C14—C13—H13	126.4
N1—Cd1—O5	81.39 (7)	N4—C13—H13	126.4
N3—Cd1—O5	170.77 (7)	C13—C14—N3	110.2 (3)
O3—Cd1—O5	84.80 (7)	C13—C14—H14	124.9
O4—Cd1—O5	89.35 (6)	N3—C14—H14	124.9
O5^i^—Cd1—C25	131.37 (9)	N3—C15—N4	112.6 (2)
N1—Cd1—C25	119.91 (10)	N3—C15—H15	123.7
N3—Cd1—C25	82.74 (8)	N4—C15—H15	123.7
O3—Cd1—C25	27.40 (9)	N4—C16—C17	111.1 (2)
O4—Cd1—C25	27.21 (8)	N4—C16—H16A	109.4
O5—Cd1—C25	88.09 (8)	C17—C16—H16A	109.4
C25—O3—Cd1	93.93 (19)	N4—C16—H16B	109.4
C25—O4—Cd1	89.92 (19)	C17—C16—H16B	109.4
C27—O5—Cd1^i^	110.77 (16)	H16A—C16—H16B	108.0
C27—O5—Cd1	133.36 (17)	C18—C17—C16	113.1 (3)
Cd1^i^—O5—Cd1	107.90 (7)	C18—C17—H17A	109.0
H7A—O7—H7B	116.4	C16—C17—H17A	109.0
H8A—O8—H8B	116.3	C18—C17—H17B	109.0
C12—N1—C11	105.6 (2)	C16—C17—H17B	109.0
C12—N1—Cd1	129.21 (18)	H17A—C17—H17B	107.8
C11—N1—Cd1	125.20 (18)	O2—C18—C19	121.2 (3)
C12—N2—C10	106.5 (2)	O2—C18—C17	120.7 (3)
C12—N2—C9	126.8 (2)	C19—C18—C17	118.0 (3)
C10—N2—C9	126.7 (2)	C20—C19—C24	118.8 (3)
C15—N3—C14	104.1 (2)	C20—C19—C18	122.9 (3)
C15—N3—Cd1	129.63 (18)	C24—C19—C18	118.3 (3)
C14—N3—Cd1	126.21 (19)	C19—C20—C21	120.7 (3)
C15—N4—C13	105.9 (2)	C19—C20—H20	119.7
C15—N4—C16	127.6 (2)	C21—C20—H20	119.7
C13—N4—C16	126.3 (2)	C22—C21—C20	119.5 (4)
C2—C1—C6	120.5 (3)	C22—C21—H21	120.3
C2—C1—H1	119.7	C20—C21—H21	120.3
C6—C1—H1	119.7	C23—C22—C21	120.2 (4)
C3—C2—C1	120.4 (3)	C23—C22—H22	119.9
C3—C2—H2	119.8	C21—C22—H22	119.9
C1—C2—H2	119.8	C22—C23—C24	120.5 (4)
C2—C3—C4	119.9 (3)	C22—C23—H23	119.7
C2—C3—H3	120.1	C24—C23—H23	119.7
C4—C3—H3	120.1	C23—C24—C19	120.3 (4)
C3—C4—C5	120.2 (3)	C23—C24—H24	119.9
C3—C4—H4	119.9	C19—C24—H24	119.9
C5—C4—H4	119.9	O4—C25—O3	121.3 (3)
C4—C5—C6	120.3 (3)	O4—C25—C26	119.0 (3)
C4—C5—H5	119.9	O3—C25—C26	119.7 (3)
C6—C5—H5	119.9	O4—C25—Cd1	62.87 (16)
C5—C6—C1	118.7 (3)	O3—C25—Cd1	58.67 (15)
C5—C6—C7	122.7 (2)	C26—C25—Cd1	173.5 (2)
C1—C6—C7	118.5 (2)	C25—C26—H26A	109.5
O1—C7—C6	120.6 (2)	C25—C26—H26B	109.5
O1—C7—C8	120.0 (2)	H26A—C26—H26B	109.5
C6—C7—C8	119.4 (2)	C25—C26—H26C	109.5
C7—C8—C9	111.6 (2)	H26A—C26—H26C	109.5
C7—C8—H8A'	109.3	H26B—C26—H26C	109.5
C9—C8—H8A'	109.3	O6—C27—O5	122.6 (2)
C7—C8—H8B'	109.3	O6—C27—C28	119.4 (3)
C9—C8—H8B'	109.3	O5—C27—C28	118.0 (3)
H8A'—C8—H8B'	108.0	C27—C28—H28A	109.5
N2—C9—C8	111.5 (2)	C27—C28—H28B	109.5
N2—C9—H9A	109.3	H28A—C28—H28B	109.5
C8—C9—H9A	109.3	C27—C28—H28C	109.5
N2—C9—H9B	109.3	H28A—C28—H28C	109.5
C8—C9—H9B	109.3	H28B—C28—H28C	109.5
H9A—C9—H9B	108.0		
			
O5^i^—Cd1—O3—C25	−165.80 (17)	C10—N2—C9—C8	−73.3 (4)
N1—Cd1—O3—C25	−29.1 (2)	C7—C8—C9—N2	175.6 (2)
N3—Cd1—O3—C25	78.93 (18)	C12—N2—C10—C11	0.1 (3)
O4—Cd1—O3—C25	−3.00 (17)	C9—N2—C10—C11	178.0 (3)
O5—Cd1—O3—C25	−95.91 (18)	N2—C10—C11—N1	−0.7 (4)
O5^i^—Cd1—O4—C25	44.4 (3)	C12—N1—C11—C10	1.1 (3)
N1—Cd1—O4—C25	168.43 (18)	Cd1—N1—C11—C10	179.5 (2)
N3—Cd1—O4—C25	−89.67 (18)	C11—N1—C12—N2	−1.1 (3)
O3—Cd1—O4—C25	3.02 (17)	Cd1—N1—C12—N2	−179.42 (16)
O5—Cd1—O4—C25	87.10 (18)	C10—N2—C12—N1	0.6 (3)
O5^i^—Cd1—O5—C27	144.8 (3)	C9—N2—C12—N1	−177.3 (2)
N1—Cd1—O5—C27	−110.7 (2)	C15—N4—C13—C14	−0.3 (4)
N3—Cd1—O5—C27	3.1 (6)	C16—N4—C13—C14	174.2 (3)
O3—Cd1—O5—C27	37.2 (2)	N4—C13—C14—N3	0.7 (5)
O4—Cd1—O5—C27	−17.2 (2)	C15—N3—C14—C13	−0.9 (4)
C25—Cd1—O5—C27	10.0 (2)	Cd1—N3—C14—C13	177.8 (3)
O5^i^—Cd1—O5—Cd1^i^	0.0	C14—N3—C15—N4	0.7 (4)
N1—Cd1—O5—Cd1^i^	104.48 (9)	Cd1—N3—C15—N4	−177.87 (18)
N3—Cd1—O5—Cd1^i^	−141.7 (4)	C13—N4—C15—N3	−0.3 (4)
O3—Cd1—O5—Cd1^i^	−107.62 (9)	C16—N4—C15—N3	−174.7 (3)
O4—Cd1—O5—Cd1^i^	−162.07 (8)	C15—N4—C16—C17	100.1 (3)
C25—Cd1—O5—Cd1^i^	−134.88 (10)	C13—N4—C16—C17	−73.3 (4)
O5^i^—Cd1—N1—C12	164.3 (2)	N4—C16—C17—C18	171.1 (2)
N3—Cd1—N1—C12	−76.8 (2)	C16—C17—C18—O2	0.8 (4)
O3—Cd1—N1—C12	26.7 (3)	C16—C17—C18—C19	−178.9 (2)
O4—Cd1—N1—C12	5.7 (2)	O2—C18—C19—C20	−178.7 (3)
O5—Cd1—N1—C12	94.5 (2)	C17—C18—C19—C20	1.0 (4)
C25—Cd1—N1—C12	11.8 (3)	O2—C18—C19—C24	1.5 (4)
O5^i^—Cd1—N1—C11	−13.7 (2)	C17—C18—C19—C24	−178.7 (3)
N3—Cd1—N1—C11	105.1 (2)	C24—C19—C20—C21	0.1 (5)
O3—Cd1—N1—C11	−151.3 (2)	C18—C19—C20—C21	−179.6 (3)
O4—Cd1—N1—C11	−172.4 (2)	C19—C20—C21—C22	0.4 (5)
O5—Cd1—N1—C11	−83.5 (2)	C20—C21—C22—C23	−0.8 (6)
C25—Cd1—N1—C11	−166.3 (2)	C21—C22—C23—C24	0.7 (6)
O5^i^—Cd1—N3—C15	25.8 (3)	C22—C23—C24—C19	−0.1 (5)
N1—Cd1—N3—C15	−82.6 (2)	C20—C19—C24—C23	−0.3 (5)
O3—Cd1—N3—C15	131.2 (2)	C18—C19—C24—C23	179.5 (3)
O4—Cd1—N3—C15	−174.2 (3)	Cd1—O4—C25—O3	−5.3 (3)
O5—Cd1—N3—C15	165.2 (3)	Cd1—O4—C25—C26	172.9 (3)
C25—Cd1—N3—C15	158.3 (3)	Cd1—O3—C25—O4	5.6 (3)
O5^i^—Cd1—N3—C14	−152.5 (3)	Cd1—O3—C25—C26	−172.7 (3)
N1—Cd1—N3—C14	99.1 (3)	O5^i^—Cd1—C25—O4	−156.24 (15)
O3—Cd1—N3—C14	−47.1 (3)	N1—Cd1—C25—O4	−13.4 (2)
O4—Cd1—N3—C14	7.5 (3)	N3—Cd1—C25—O4	86.62 (18)
O5—Cd1—N3—C14	−13.1 (6)	O3—Cd1—C25—O4	−174.6 (3)
C25—Cd1—N3—C14	−20.0 (3)	O5—Cd1—C25—O4	−92.28 (17)
C6—C1—C2—C3	0.0 (5)	O5^i^—Cd1—C25—O3	18.4 (2)
C1—C2—C3—C4	0.2 (5)	N1—Cd1—C25—O3	161.30 (16)
C2—C3—C4—C5	−0.5 (5)	N3—Cd1—C25—O3	−98.73 (18)
C3—C4—C5—C6	0.8 (5)	O4—Cd1—C25—O3	174.6 (3)
C4—C5—C6—C1	−0.6 (4)	O5—Cd1—C25—O3	82.37 (17)
C4—C5—C6—C7	−179.9 (3)	O5^i^—Cd1—C25—C26	95 (2)
C2—C1—C6—C5	0.2 (4)	N1—Cd1—C25—C26	−122 (2)
C2—C1—C6—C7	179.6 (3)	N3—Cd1—C25—C26	−22 (2)
C5—C6—C7—O1	−179.0 (3)	O3—Cd1—C25—C26	77 (2)
C1—C6—C7—O1	1.7 (4)	O4—Cd1—C25—C26	−108 (2)
C5—C6—C7—C8	0.2 (4)	O5—Cd1—C25—C26	159 (2)
C1—C6—C7—C8	−179.1 (3)	Cd1^i^—O5—C27—O6	8.6 (3)
O1—C7—C8—C9	−3.1 (4)	Cd1—O5—C27—O6	−135.5 (2)
C6—C7—C8—C9	177.7 (2)	Cd1^i^—O5—C27—C28	−170.7 (2)
C12—N2—C9—C8	104.2 (3)	Cd1—O5—C27—C28	45.2 (4)

Symmetry codes: (i) −*x*+1, −*y*+2, −*z*+2.

### Hydrogen-bond geometry (Å, °)

**Table:**

*D*—H···*A*	*D*—H	H···*A*	*D*···*A*	*D*—H···*A*
O7—H7A···O3^ii^	0.85	2.11	2.949 (3)	169
O7—H7B···O4^iii^	0.85	1.99	2.829 (3)	171
O8—H8B···O6^iii^	0.85	2.03	2.838 (3)	159
O8—H8A···O7^iv^	0.85	1.97	2.818 (3)	174

Symmetry codes: (ii) −*x*+1, *y*−1/2, −*z*+3/2; (iii) *x*, *y*, *z*−1; (iv) −*x*+1, −*y*+1, −*z*.
